# Anaphylactic Shock Caused by Eating Buckwheat

**DOI:** 10.3390/jcm13175243

**Published:** 2024-09-04

**Authors:** Dan Orga-Dumitriu, Dana M. Harris, Corina Porr

**Affiliations:** 1 Internal Medicine Department, Faculty of Medicine, Lucian Blaga University, 550169 Sibiu, Romania; danorgadumitriu@yahoo.com; 2 Internal Medicine Department, Mayo Clinic, Jacksonville, FL 32224, USA; danamcharris@yahoo.com; 3 Allergology Department, County Emergency Clinical Hospital, 550245 Sibiu, Romania

**Keywords:** hidden allergen, anaphylactic shock, hives, food, buckwheat

## Abstract

**Background**: Urticaria is a common disease with a marked influence on quality of life. The key cell involved is the mast cell, which can be activated by a vast variety of stimuli, and the major mediator is histamine. Allergic urticaria is a disorder with a large variety of causes: food, drugs, insect venom, skin contact with allergens, and physical exercise. Buckwheat consumption has increased in European countries and the USA because it is gluten-free. It can trigger anaphylactic shock if ingested, inhaled, or handled with the hands. Five common buckwheat allergens named Fag e1 to 5 (Fag e1, 2, and 3 are considered the major allergens) and two tartary buckwheat allergens named Fag t1 and Fag t2 have been described. **Method**: We present the case of a patient who experienced two anaphylactic shocks and in whom the etiological factor was buckwheat. The patient presented to the Allergology department for the evaluation of two episodes of severe allergic reactions that required emergency therapy, episodes that involved the loss of consciousness and were of major severity. At each anaphylactic shock, an ambulance was requested, and emergency therapy was administered, leading to the patient’s recovery within a few hours. **Diagnosis**: Since each episode occurred a few minutes after eating, the diagnosis was established based on a detailed anamnesis and prick skin tests, followed by specific IgE dosages. Other foods consumed by the patient, assessed by prick skin testing and specific IgE dosages of suspected foods, were excluded as the etiological cause. Increased levels of buckwheat-specific immunoglobulin E were highlighted, thus identifying the etiological agent. The treatment of anaphylactic shock was performed urgently by the ambulance crew with adrenaline, infusion solutions, cortisone preparations, and antihistamines. **Result**: Following the treatment that was initiated, there was a partial remission of the lesions after a few hours. **Conclusions**: Buckwheat allergy is rare, but it produces symptoms that affect the skin, gastrointestinal tract, and respiratory tract, as well as anaphylaxis. In a professional environment, it can trigger allergic rhinitis, asthma, and hives. Although buckwheat allergens have been described, their clinical relevance has only been studied in a small number cases. In current practice, the only commercially available allergen is Beech e2 per the ImmunoCAP ISAC microarray. Diagnosis can be difficult in clinical practice. This reported case suggests the need for a thorough anamnesis, since buckwheat is consumed as a hidden allergen, and in Europe, it is not necessary to label foods containing this allergen.

## 1. Introduction

In recent years, the incidence of food allergies has increased a lot and is a major health problem [1]. Between 3 and 10% of children are affected, and the incidence of food allergies in adults can reach up to 10% [2,3,4]. Food allergy is an adverse reaction to food that is mediated by the immune system, where a protein in the structure of the food is recognized as foreign by the body. The most common foods that cause food allergies are shellfish, fish, peanuts, cow’s milk, soy, wheat flour, and nuts, which are responsible for more than 90% of food allergies. The first allergies that occur in children are caused by the protein in cow’s milk and eggs, which disappear around the age of 3 in most children, but they are at risk of developing respiratory allergies during adolescence. In adults, the most common foods include cereals, which are indispensable foods in our diet, and are another important category of foods that can cause allergies, the most common being wheat flour, but barley, rye, and corn flour can also be implicated. These are a group of plants that are part of the Gramineae family, and their consumption can prevent the onset of cardiovascular diseases. Cereals are staple foods, rich in fiber and carbohydrates, that help the body develop and function properly. They are introduced into the diet of rams during diversification, starting at the age of 6 months. Rice is a grain that has a low risk of causing allergies but contains the eight essential amino acids. Millet is very easily digested, contains magnesium, iron, calcium, and B vitamins, and has a low glycemic index, making it useful for those with diabetes. Quinoa is rich in protein and contains thiamine, niacin, riboflavin, pantothenic acid, vitamin B-6, folic acid, and vitamin E. It also contains minerals such as calcium, iron, phosphorus, potassium, magnesium, sodium, and zinc. Amaranth, another grain, is gluten-free, easy to digest, and rich in protein, fiber, vitamin E, calcium, magnesium, phosphorus, iron, and manganese. Barley is a food that is relatively easy to digest and contains fiber, phosphorus, amino acids, iron, copper, B vitamins, and manganese. Buckwheat is rich in iron, fiber, vitamin K, and folic acid and can be introduced into a baby’s diet starting from the age of 6 months. You can use boiled and mashed grains, flour, or buckwheat cereals. Corn is gluten-free, but it does contain vitamin B1, vitamin E, iron, phosphorus, magnesium, zinc, and small amounts of manganese and copper. Oats belong to the category of cereals with gluten, but the amount of gluten is lower than in the case of wheat, so the allergenic risk is lower compared to wheat. They contain fiber, calcium, manganese, zinc, selenium, B vitamins, vitamin E, and vegetable proteins. Flour or cereal dust, if inhaled, can trigger rhinitis or asthma. If ingested, they can cause celiac disease, food allergies, and dermatitis herpetiformis [5].

Epidemiological data are variable, and most refer to wheat allergy, which is the best known and studied. Allergies induced by other grains are uncommon. IgE-mediated wheat allergy affects 0.2–1.3% of children in Europe and 0.4% of children in the United States. Epidemiological data for other wheat-like cereals are largely lacking. A study conducted in a pediatric outpatient clinic specializing in food allergies reveals a prevalence of wheat allergy of 18% in the pediatric population studied [6]. The real prevalence of buckwheat allergy is not known. The highest prevalence of buckwheat allergy is in Asia, at 0.1% in the Republic of Korea and 0.22% in Japan. It is worth noting that in Japan, it is the sixth most common cause of food allergy and causes about 3% of food anaphylaxis, and in a study that included school-age children, more than half had experienced anaphylaxis. There is an increase in the prevalence of this allergy in patients with celiac disease. The prevalence in European countries and the United States ranges from 1% to 9.7%. We will present the case of a patient who had a severe allergic reaction after eating pizza.

## 2. Case Report

A 43-year-old male patient from an urban area presented to the allergy clinic for the evaluation of two episodes of severe allergic reactions that required emergency therapy, episodes that involved the loss of consciousness and were of major severity. Hereditary collateral history is unimportant for the current condition. The patient has had manifestations of allergic rhinitis since the age of 20, but the diagnosis of moderate/severe persistent allergic rhinitis was at 25 years of age. The patient is a non-smoker and consumes alcohol occasionally. The patient has a bakery and has been working in the bakery industry for 10 years.

In 2020, the patient ate pizza in a restaurant, and 10 min later, he complained of a burning sensation on his tongue, followed by erythema of the ears and hands. The patient ate pizza that contained cereal dough, with the following ingredients added: mozzarella, tomatoes, and champignon mushrooms. A few minutes later, he complained of the appearance of erythematous macules and papules on his trunk, followed by edema of the tongue. An ambulance was requested, and at the time of arrival, the patient was in an altered general condition with BP = 80/50 mmHg. Adrenaline therapy, infusion solutions, and cortisone preparations were administered.

A month after this event, a few minutes after consuming Kürtőskalács (a Hungarian spit cake), the patient complained of intense itching in his tongue and oral cavity, but this time, the patient quickly requested an ambulance, as he was sensitized from the previous incident. The Kürtőskalács was made with wheat flour and buckwheat flour and covered with sugar. Before the ambulance arrived, he had a dry cough, dyspnea mixed with wheezing, an erythematous rash with itchy macules on his trunk, and generalized skin pruritus, and at the arrival of the ambulance, the patient had a BP of 80/50 mmHg. Adrenaline therapy, cortisone preparations, infusion solutions, and calcium preparations were administered, and he was again admitted to the emergency unit. The patient was stabilized and again requested to leave the hospital with the recommendation of antihistamine therapy at home and an allergy consultation.

Three weeks after the second incident described above, the patient went to see an allergologist. The pharmacist recommended desloratadine 5 mg on a need basis for allergic rhinitis. At the time of presentation at the allergologist, the physical examination was normal (SaO_2_ = 99%, BP = 120/70 mmHg, VA = 70/min). Based on that and the patient’s history, a diagnosis of mild intermittent allergic rhinitis and grade III anaphylactic reaction after food consumption was established.

Prick allergy skin tests were performed on environmental and food allergens. The environmental allergens with which the prick skin tests were performed were Dermatophagoides pteronissinus and farinae, grass pollen, betulaceae (birch, hazelnut, alder), ambrosia artemisiifolia, artemisia vulgaris, cladosporium herbarum, and dog and cat epithelium, with the negative control and positive control being histamine. Skin tests performed with standardized allergenic extracts (Alyostal, Stallergens, Antony, France) revealed moderate skin sensitization to grass pollen (papule 6 mm). Skin tests for food allergens (tomatoes, mushrooms, wheat flour, egg yolk, egg white, chicken, pork, beef, milk, apple, peach, banana, peanuts, nuts, fish) were negative. Please note that a buckwheat prick allergen test was not available. Prick-to-prick testing on buckwheat was discussed, but it was not available at the time of testing. The respiratory functional tests performed at the first presentation in the allergy department were within normal limits.

The allergologist further recommended determining the level of serum-specific IgE on different grains because the Kürtőskalács was made from mixed-grain flour. The patient received the recommendation to continue taking desloratadine as needed and to permanently wear an adrenaline autoinjector, Epipen 03.mg, so that he could administer it when necessary. During the doctor’s visit, the patient was instructed on how to use the Epipen injector.

The patient returned after a month for a new evaluation. During this period, the patient did not eat out (such as dining at restaurants) and did not show any pathological manifestations after meals. The serum IgE tests performed before this doctor’s visit revealed an increased level (5.7 KU/L) for buckwheat. Fag e2 in the ImmunoCAP ISAC microarray was available for testing; we performed it, and the value was increased.

The doctor recommended avoiding the consumption of buckwheat and preparations containing this cereal, given that it can be hidden in various pastry preparations, and continuing with wearing the adrenaline autoinjector.

After two years, the patient returned to the allergologist to complain of daily episodes of dyspnea, wheezing, dry cough in attacks, fatigue, and a sensation of chest compression that lasted for approximately two weeks. The symptoms started four months prior to the doctor’s visit, initially appearing rarely, not all at once, but progressively intensified, becoming daily. He had no more anaphylactic or skin reactions since the last presentation, only intermittent nasal symptoms which were controlled with antihistamines as needed.

On physical examination, at chest auscultation, bilaterally disseminated wheezing rales were revealed, with BP = 120/70 mmHg, VA = 79/min, and SaO_2_ = 98%. Functional respiratory tests were performed that showed moderate obstructive ventilatory dysfunction, and then, a bronchodilation test with salbutamol (two puffs) was performed, yielding a positive result after 20 min. The diagnosis of mild intermittent allergic rhinitis, newly diagnosed allergic asthma, and Muller III anaphylactic reaction was established. Therapy was initiated with a combination of budesonide and formoterol 160/4.5 μg, with two puffs/day and one additional puff on a need basis, with rinsing of the mouth after use. He was advised to return in two weeks to be evaluated.

The patient returned to the allergy department after six months. During this period, the patient used the prescribed therapy only as needed, several times a week, in the form of one puff of the combination of formoterol and budesonide. He had daily symptoms, with nocturnal awakenings and the inability to rest, leading to an impairment of quality of life and work capacity. The physical examination revealed wheezing rales and facies, but normal blood pressure values, pulse, and oxygen saturation.

Spirometry revealed mixed ventilatory dysfunction. An asthma control test (ACT) questionnaire was conducted, and an 8/25 score was obtained. The follow-up recommendation was to perform a blood count, inflammatory sampling, pharyngeal and nasal exudate collection, and lung X-ray. Blood eosinophilia was revealed by these tests. Chest X-ray indicated a bilaterally accentuated interstitial pattern with no areas of pulmonary condensation.

The diagnosis established that this was mild intermittent allergic rhinitis, severe persistent allergic asthma (stage IV treatment), and Muller III anaphylactic reaction to buckwheat. A new therapeutic protocol was prescribed composed of formoterol and budesonide 160/4.5 μg, two puffs twice daily, with rinsing of the mouth after use, montelukast 10 mg each evening, and desloratadine 5 mg/day, on a need basis, in the presence of nasal symptoms. Since the patient was non-compliant, it was strongly recommended that he return for a check-up after a month. After a week, the patient called to inform the doctor that his general condition had improved significantly.

At the one-month check-up, the physical examination did not reveal the presence of wheezing rales, and the patient reported no symptoms during the therapy. However, when the patient missed a dose inadvertently, the symptoms reappeared, and he had to administer his medication. The spirometry performed was within normal limits. He was recommended to continue the prescribed therapy and return for a check-up after two months. For the next two years, he returned for check-ups twice, and during this time, the patient was very non-compliant. The immediate prognosis was favorable in the short term, but the patient’s non-compliance led to an unfavorable prognosis and the evolution of uncontrolled asthma. Due to the patient’s lack of compliance, we insisted that he come back for follow-up. Even if the short-term evolution may be favorable, it can be maintained in the long term if the patient continues his therapy. Daily use of medication for bronchial asthma and allergic rhinitis leads to control of the diseases and prevents their progression. Periodic assessment of lung function by spirometry is also useful. If the patient does not follow the prescribed therapy and does not present for periodic evaluation, he/she may develop complications, such as otitis, sinusitis, sleep disturbances, spontaneous pneumothorax, bronchiectasis, and chronic pulmonary cords.

## 3. Discussion

The prevalence of food allergies is constantly increasing, and as far as cereal allergy is concerned, there are numerous studies on wheat flour and much less on other cereals, namely buckwheat. Data related to buckwheat allergy do not exist in Romania, only case reports. Buckwheat is frequently used by people who do not consume gluten, and lately, its consumption has increased in Europe and the USA. It is used in many foods in the form of porridge, pastries, noodles, pancakes, and bread. Buckwheat husks are also used to fill pillows, and in this case, allergic reactions occur by inhalation. This is a pseudocereal that belongs to the group of the Polygonaceae family. Allergenic buckwheat proteins are seed-storing proteins that belong to the group of the prolamin and cuprin superfamilies [7].

Worldwide, the World Health Organization (WHO) has reported five common buckwheat allergens, Beech e1–5, and two tartary buckwheat allergens, Beech t1–2. Beech e1, e2, and e3 are considered major allergens [8]. In the case presented here, we deal with an atopic adult patient with manifestations of allergy to grass pollen and also buckwheat. Although the buckwheat allergy is rare, it can cause anaphylactic reactions, as it is consumed as a hidden food allergen. Only in the Republic of Korea and Japan is there a labeling regulation for this allergen [9].

Allergic reactions to buckwheat can occur through ingestion, inhalation, or handling. The clinical manifestations are those typical of food allergies, with skin, gastrointestinal tract, and respiratory tract symptoms, but also anaphylaxis [10]. Allergic rhinitis, asthma, and contact urticaria have been described as occupational diseases [11]. Sensitization to Beech e2, which is an important allergen, is frequently associated with anaphylactic manifestations [12,13,14].

To establish the diagnosis of buckwheat allergy, in addition to the anamnesis, we can perform prick skin testing or tests with specific IgE dosages for buckwheat, but the specificity of these tests is low and the sensitivity is high. Five common buckwheat allergens named Fag e1 to 5 (Fag e1, 2, and 3 are considered the major allergens) and two tartary buckwheat allergens named Fag t1 and Fag t2 have been described. Beech e2 from the ImmunoCAP ISAC microarray is the only commercially available allergen for the diagnosis of this allergy. But even if it is not increased, we cannot exclude this allergy, because it can also be sensitized by other allergens (Beech is 1,3,5). Sensitization to Fag e2 is often related to severe reactions including anaphylaxis, and it is thus considered an important allergen in buckwheat anaphylaxis. If the patient does not have an anaphylactic reaction, but we suspect this allergy, the recommendation is to perform an oral food challenge test. Since prick skin tests and serum IgE dosages for the offending foods were excluded, buckwheat remained in question as a triggering allergen. Since there is no standardized allergen for prick testing and prick-to-prick testing could not be performed, we resorted to the dosage of serum IgE specific to buckwheat. Although buckwheat allergens have been described, their clinical relevance has only been studied in a small number of cases. In current practice, the only commercially available allergen is Beech e2 per the ImmunoCAP ISAC microarray. Diagnosis can be difficult in clinical practice due to the impossibility of dosing all allergens. It would be useful to be able to determine all buckwheat allergens and to be able to study their clinical relevance in as many patients as possible.

In the case reported here, no oral buckwheat challenge test was performed because the patient had anaphylaxis. For the other cereals, the specific IgE dosages were within normal limits, and it was not necessary to exclude them from the diet. The symptoms of buckwheat allergy in this patient were cutaneous and respiratory, finally reaching anaphylaxis. The reactions in this case were severe and required the administration of adrenaline, but the symptoms remitted with the administration of medication.

In the emergency department, it is not possible to dose tryptase. It would have been very useful to be able to determine its value during anaphylactic shock. The measurement was performed 2 weeks after the event, and was within normal limits. This determination was made in a private laboratory at our indication, and the cost was borne by the patient, as it is not included in medical insurance in our country. Regarding possible mastocytosis, we determined 2 weeks after anaphylactic shock that the c-kit mutation D816V was negative. We should mention that this determination is not supported by medical insurance and the cost was also borne by the patient.

The management of grain and buckwheat allergy includes measures specific to any food allergy. Short-term measures are needed to treat an acute reaction, and long-term measures are needed to reduce the risk and prevent other acute reactions. Emergency treatment is recommended if the patient is accidentally exposed to buckwheat, as it can often be a hidden allergen. Avoidance measures should be maintained both in the short and long term. Anaphylaxis may also recur if this food is eaten after a longer break. Both short-term and long-term hand contact should also be avoided, as you may develop skin symptoms initially, but if there is re-exposure, you may develop anaphylaxis through skin contact. Through inhalation, if he/she has contact with hemp, the initial manifestation may be dyspnea, but then, respiratory symptoms may worsen. Buckwheat flour can be substituted with wheat flour, for example, but also with other types of cereal flours, as the patient is not sensitized to them.

Treatment of allergic rhinitis to pollen during the pollen season was also recommended. The patient presented in this case also suffered from bronchial asthma, hence the recommendation to evaluate its severity level at each medical visit. However, with a non-compliant patient like the one described here, there are additional challenges to keeping the allergy under control. In such cases, we can integrate biological therapy into the treatment regimen.

The risk of an acute reaction varies between patients who have a food allergy, and patients who have a history of anaphylactic reactions or who also have uncontrolled bronchial asthma, but also those who have certain cofactors such as physical effort, taking anti-inflammatory drugs, or mastocytosis, have an increased risk of severe reactions. In this case, the patient had cofactors such as bronchial asthma and the two anaphylactic episodes, indicating an increased risk of developing severe allergic reactions induced by buckwheat.

For the successful long-term treatment of food allergies, the patient education component is critical. The first step is to eliminate the offending food from the diet. A dietitian can advise the family on how to avoid the offending food, including when hidden in different dishes. The patient can find out information about this food by reading the labels on food products. This is, unfortunately, not possible in European countries or the US. In these countries, buckwheat is not included in the list of food allergens on product labels according to the Consumers Regulation (EU) No. 1169/2011 (FDA) [15]. These can, therefore, cause severe reactions and anaphylactic manifestations as buckwheat is present as a hidden allergen [16].

The particularity of the case we reported here lies in the fact that the patient had a severe food allergy in adulthood, with an atopic terrain and intermittent allergic rhinitis. The allergist–dietician–nurse–patient team is very important in order to recommend a correct exclusion diet that avoids accidental exposure to the offending food.

## Data Availability

The datasets used and/or analyzed during the current study are available from the corresponding author on reasonable request.
